# Supplementary material for: The Modification of the Illumina® CovidSeq™ Workflow for RSV Genomic Surveillance: The Genetic Variability of RSV during the 2022–2023 Season in Northwest Spain
Source: Int J Mol Sci. 2023 Nov 7;24(22):16055. doi: 10.3390/ijms242216055 (PMC10671726; doi:10.3390/ijms242216055)
Supplement: Supplementary file 1 [file ijms-24-16055-s001.zip › Supplementary figures.pdf]

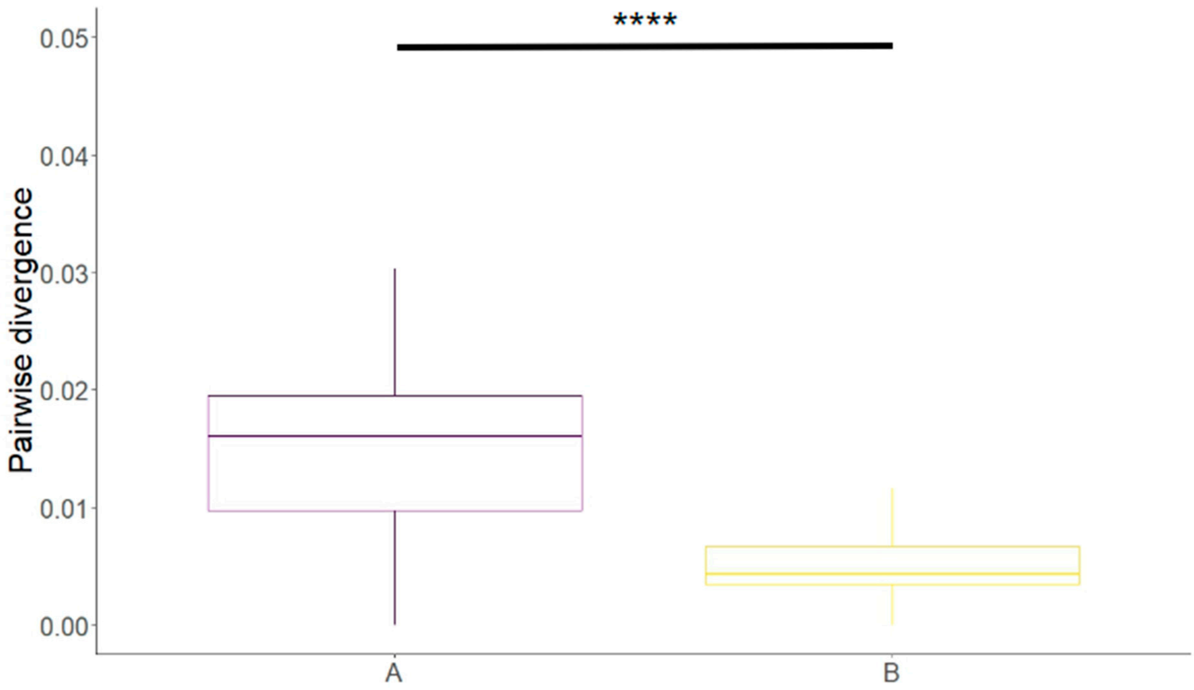

Supplementary Figure S1: RSV-A showed more genetic diversity than RSV-B in the European samples of 2022-2023. Hamming distance was calculated for all pairwise combinations between RSV-A samples or between RSV-B samples. Distance is significantly higher for RSV-A (Wilcoxon rank-sum test, $p < 0.0001$). Outliers were removed from the graph to facilitate visualization.

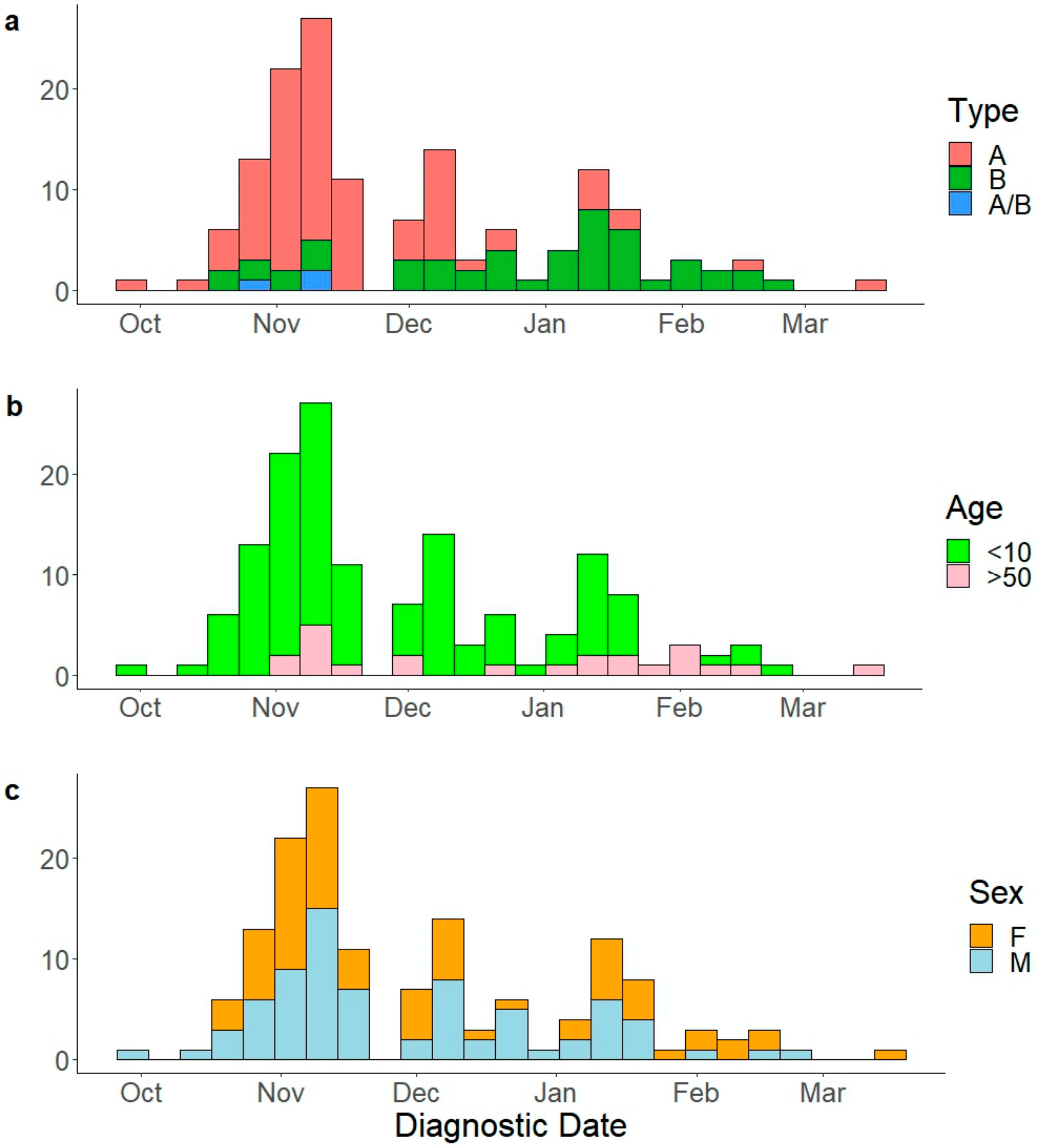

Supplementary Figure S2: characteristics of the infected population of the sequences in this study. (**a**) Population by subtype. RSV-A was more common during the first months of the period, while RSV-B was more common towards the end. (**b**) Population by age. The majority of the population studied was pediatric, with 84.3% of the individuals being below 10 y/o. (**c**) Population by sex. Population equally distributed by sex (49.0 %; 51.0 %)

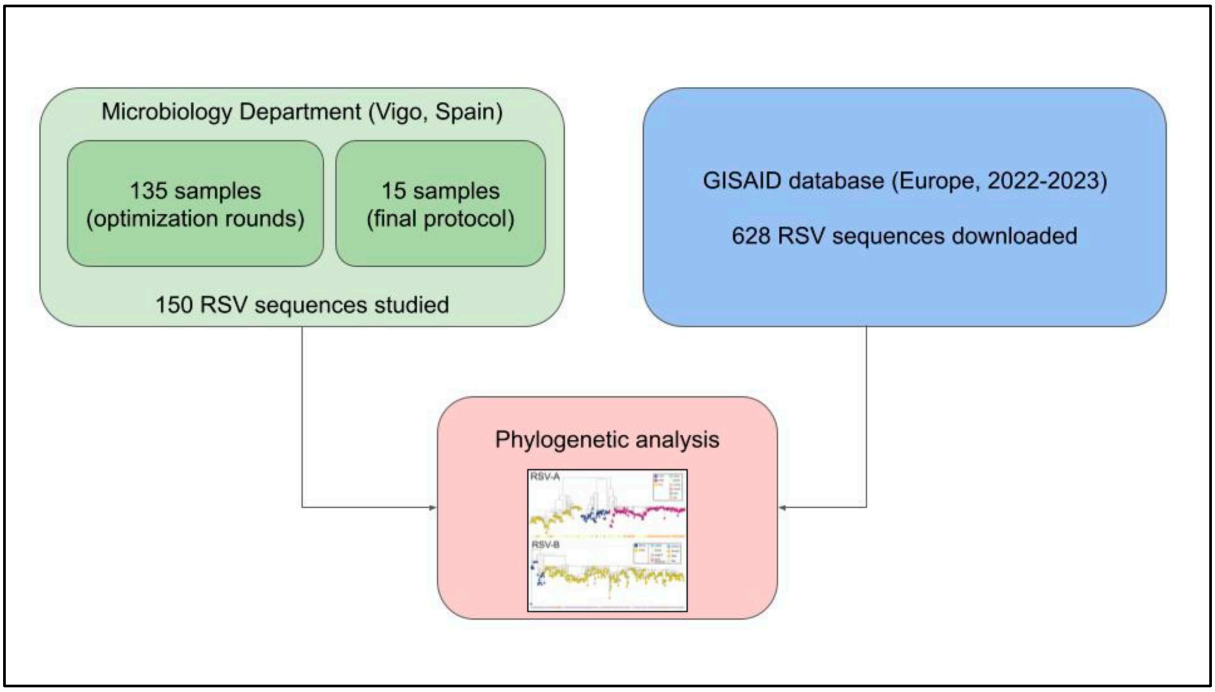

Supplementary Figure S3: description of the source of all samples used in this study.
