# Supplementary material for: The Modification of the Illumina® CovidSeq™ Workflow for RSV Genomic Surveillance: The Genetic Variability of RSV during the 2022–2023 Season in Northwest Spain
Source: Int J Mol Sci. 2023 Nov 7;24(22):16055. doi: 10.3390/ijms242216055 (PMC10671726; doi:10.3390/ijms242216055)
Supplement: Supplementary file 1 [file ijms-24-16055-s001.zip › Supplementary Table S1.pdf]

Supplementary Table S1: Primers used for RSV-A and RSV-B amplification. Volumes in microliters of 100uM stock concentration primers. To create a 10uM working concentration, 1017ul and 1035ul of nuclease-free water were added to pool 1 and 2 respectively. For references, HRSV/A/England/397/2017 and HRSV/B/Australia/VIC-RCH056/2019 were used for RSV-A and B respectively.

Primer	Volume	Source	Sequence	Location	Pool
A1f	5	Wang [16]	ACGSGAAAAAATGCGTACAAC	1	1
A1r	5	Wang	GAAGATTGTGCTATACCAAAATGAACA	1779	1
AB3f	10	Goya [3]	GCYATGGCAAGACTYAGGAATG	2897	1
A3r	5	Wang	GTTTGCYGAGGCTATGAATATGAT	4826	1
A5f	5	Wang	GAACAACAGACTACTAGAGATTACCAG	6374	1
A5r	10	This publication	AGGAGTTTRCTCATRGCAA	7929	1
A7f	5	Wang	AGCTTAGGCTTAAGATGYGGA	9423	1
A7r	5	Wang	TGAGTTTGACCTTCCATGAGT	10997	1
A9f	7	Wang	GGGTTGGTTCATCTACACAAGAG	12316	1
A9r	7	This publication	CGCAATAATAAATTCCCTGCTCC	14094	1
B1f	5	Wang	ACGCGAAAAAATGCGTACTACA	1	1
B1r	5	Wang	CATTGTTTGCCCTCCTAATTACTG	1661	1
B3r	5	Wang	ATAGGGCCAAAATTTGCTTGTG	4309	1
B5f	5	Wang	AGTGCAATCTTCCTAACTCTTGC	5700	1
B5r	5	Wang	TGATTCCACTTAGTTGGTCTTTGC	7375	1
B7f	5	Wang	GGTGAACTGAAATTAGAAGAACCAAC	8760	1
B7r	5	Wang	CACCATATCTTGTCAAACTCTCAGG	10507	1
B9f	7	Wang	GAACCAACTTACCCTCATGGATT	11860	1
B9r	7	Wang	TTCTGGGGTTGGGTGATATAG	13650	1
A2f	5	Wang	ACAGGCATGACTCTCCTGAT	1556	2
A2r	5	Wang	TTGGGTGTGGATATTTGTTTCAC	3400	2
A4f	5	Wang	ACCTGGGACACTCTCAATCA	4697	2
A4r	5	Wang	GACATGATAGAGTAACTTTGCTGTCT	6540	2
A6f	5	Wang	GTCACGAAGGAATCCTTGCA	7642	2
A6r	5	Wang	CCCTCTACCTCTTTTATTATGTAGAACC	9521	2
A8f	5	Wang	GGTGTACAATCTCTATTTTCCTGGT	10704	2
A8r	5	Wang	CGATTAATAGGGCTAGTATCAAAGTG	12615	2
A10f	10	This publication	CRTCTACAATGATTAGAACCAATTAC	13742	2
A10r	10	Wang	ACGAGAAAAAAAGTGTCAAAAACTAA	15225	2
B2f	5	Wang	CAGRTTAGGAAGGGAAGACACTA	1316	2
B2r	5	Wang	CAAGTCACTCAATTTTTTGGAGGTTGG	2982	2
B4f	10	Wang	TGGAAGCAYACAGCTACACG	3943	2
B4r	10	Wang	CTACATGTYGATTGGTAAAACTCC	5788	2
B6f	5	Wang	CCTCTAGTGTTTCCTTCTGATGAG	7113	2
B6r	5	Wang	GTTGTAGCAATTTGTTCAGACGAG	8834	2
B8f	5	Wang	AAGTTCTCTGAAAGCGACAGATC	10231	2
B8r	5	This publication	TAATACTWGGTGATGTTACTCCTAC	12190	2
B10f	5	Wang	TAGTCAATCAAGACACAAGTTTGC	13289	2
B10r	5	Wang	ACGAGAAAAAAAGTGTCAAAAACTAATG	15222	2

References

[16] Wang L, Ng TFF, Castro CJ, Marine RL, Magaña LC, Esona M, et al. Next-generation sequencing of human respiratory syncytial virus subgroups A and B genomes. J Virol Methods 2022;299:114335. https://doi.org/10.1016/j.jviromet.2021.114335.

[3] Goya S, Rojo GL, Jordar MSN, Valinotto LE, Mistchenko AS, Viegas M. Whole genome sequencing of respiratory syncytial (RSV) virus from clinical samples with low viral load 2018. https://doi.org/dx.doi.org/10.17504/protocols.io.bmhak32e.
