# Supplementary material for: The Modification of the Illumina® CovidSeq™ Workflow for RSV Genomic Surveillance: The Genetic Variability of RSV during the 2022–2023 Season in Northwest Spain
Source: Int J Mol Sci. 2023 Nov 7;24(22):16055. doi: 10.3390/ijms242216055 (PMC10671726; doi:10.3390/ijms242216055)
Supplement: Supplementary file 1 [file ijms-24-16055-s001.zip › Supplementary Table S2.pdf]

Supplementary Table S2: Accession IDs for all samples sequenced for the study. Samples sequenced to evaluate the final protocol presented in the publication are labeled as final protocol, while samples sequenced using previous protocols in the optimization iterations are labeled as optimization run.

GISAID accesion ID	Subtype	Sequencing
EPI_ISL_16251635	A	final protocol
EPI_ISL_16251661	A	final protocol
EPI_ISL_18204850	A	final protocol
EPI_ISL_18204788	A	final protocol
EPI_ISL_16289047	A	final protocol
EPI_ISL_18204886	A	final protocol
EPI_ISL_18204829	B	final protocol
EPI_ISL_18204867	A	final protocol
EPI_ISL_18204880	B	final protocol
EPI_ISL_18204816	B	final protocol
EPI_ISL_18204842	B	final protocol
EPI_ISL_18204876	B	final protocol
EPI_ISL_16289039	A	final protocol
EPI_ISL_18204817	B	final protocol
EPI_ISL_18204854	B	final protocol
EPI_ISL_16251631	A	optimization run
EPI_ISL_16251632	A	optimization run
EPI_ISL_16251633	A	optimization run
EPI_ISL_18204865	B	optimization run
EPI_ISL_16251634	A	optimization run
EPI_ISL_18204754	A	optimization run
EPI_ISL_16251637	B	optimization run
EPI_ISL_16251638	A	optimization run
EPI_ISL_16251639	A	optimization run
EPI_ISL_16251640	A	optimization run
EPI_ISL_16251641	A	optimization run
EPI_ISL_16251643	A	optimization run
EPI_ISL_16251644	A	optimization run
EPI_ISL_16251645	B	optimization run
EPI_ISL_16251646	A	optimization run
EPI_ISL_16251647	A	optimization run
EPI_ISL_16251648	B	optimization run
EPI_ISL_16251649	B	optimization run
EPI_ISL_16251650	A	optimization run
EPI_ISL_16251651	A	optimization run
EPI_ISL_16251652	A	optimization run
EPI_ISL_16251653	A	optimization run
EPI_ISL_16251654	A	optimization run
EPI_ISL_18204771	A	optimization run
EPI_ISL_16251656	A	optimization run
EPI_ISL_16251657	A	optimization run
EPI_ISL_16251658	A	optimization run
EPI_ISL_16251662	A	optimization run
EPI_ISL_16251663	A	optimization run
EPI_ISL_16251629	A	optimization run
EPI_ISL_16251664	A	optimization run
EPI_ISL_18204780	A	optimization run
EPI_ISL_16289038	A	optimization run

EPI_ISL_16289045	A	optimization run
EPI_ISL_16251665	A	optimization run
EPI_ISL_16251666	B	optimization run
EPI_ISL_16251667	B	optimization run
EPI_ISL_16289048	A	optimization run
EPI_ISL_16289025	A	optimization run
EPI_ISL_16251668	A	optimization run
EPI_ISL_16289029	A	optimization run
EPI_ISL_18204846	A	optimization run
EPI_ISL_16289022	A	optimization run
EPI_ISL_16251669	B	optimization run
EPI_ISL_16251670	B	optimization run
EPI_ISL_16251671	A	optimization run
EPI_ISL_16289031	A	optimization run
EPI_ISL_16251672	A	optimization run
EPI_ISL_16289043	A	optimization run
EPI_ISL_16289042	A	optimization run
EPI_ISL_16251673	A	optimization run
EPI_ISL_16251674	B	optimization run
EPI_ISL_16251676	A	optimization run
EPI_ISL_16251677	A	optimization run
EPI_ISL_16251678	A	optimization run
EPI_ISL_16289031	A	optimization run
EPI_ISL_16289051	B	optimization run
EPI_ISL_18204840	A	optimization run
EPI_ISL_18277066	A	optimization run
EPI_ISL_16251680	A	optimization run
EPI_ISL_16251681	B	optimization run
EPI_ISL_16289037	A	optimization run
EPI_ISL_16251682	A	optimization run
EPI_ISL_16251683	A	optimization run
EPI_ISL_16289030	A	optimization run
EPI_ISL_16251684	A	optimization run
EPI_ISL_16251630	A	optimization run
EPI_ISL_16289041	A	optimization run
EPI_ISL_16289023	A	optimization run
EPI_ISL_16289044	A	optimization run
EPI_ISL_16289026	A	optimization run
EPI_ISL_16289032	A	optimization run
EPI_ISL_16289020	A	optimization run
EPI_ISL_16289024	A	optimization run
EPI_ISL_16289033	A	optimization run
EPI_ISL_16251685	A	optimization run
EPI_ISL_18204805	B	optimization run
EPI_ISL_16289034	A	optimization run
EPI_ISL_16289053	B	optimization run
EPI_ISL_18204773	A	optimization run
EPI_ISL_18204775	A	optimization run
EPI_ISL_16289028	A	optimization run
EPI_ISL_18204869	A	optimization run
EPI_ISL_18204848	A	optimization run
EPI_ISL_16289050	B	optimization run
EPI_ISL_16289027	A	optimization run
EPI_ISL_16289036	A	optimization run

EPI_ISL_18204786	A	optimization run
EPI_ISL_18204777	A	optimization run
EPI_ISL_18204747	A	optimization run
EPI_ISL_18204782	A	optimization run
EPI_ISL_18204838	A	optimization run
EPI_ISL_18204859	B	optimization run
EPI_ISL_18204863	B	optimization run
EPI_ISL_18204803	B	optimization run
EPI_ISL_18204794	B	optimization run
EPI_ISL_18204871	B	optimization run
EPI_ISL_18277067	A	optimization run
EPI_ISL_18204784	A	optimization run
EPI_ISL_18204809	B	optimization run
EPI_ISL_18204827	B	optimization run
EPI_ISL_18204752	B	optimization run
EPI_ISL_18204836	B	optimization run
EPI_ISL_18204792	B	optimization run
EPI_ISL_18204861	A	optimization run
EPI_ISL_18204823	B	optimization run
EPI_ISL_18204763	A	optimization run
EPI_ISL_18204831	B	optimization run
EPI_ISL_18204765	A	optimization run
EPI_ISL_18204758	A	optimization run
EPI_ISL_18204811	B	optimization run
EPI_ISL_18204767	A	optimization run
EPI_ISL_18204769	B	optimization run
EPI_ISL_18204833	B	optimization run
EPI_ISL_18204807	B	optimization run
EPI_ISL_18204882	B	optimization run
EPI_ISL_18277070	B	optimization run
EPI_ISL_18204884	B	optimization run
EPI_ISL_18277071	B	optimization run
EPI_ISL_18277068	A	optimization run
EPI_ISL_18277072	B	optimization run
EPI_ISL_18277073	B	optimization run
EPI_ISL_16289046	A	optimization run
EPI_ISL_16251628	A	optimization run
EPI_ISL_16289021	A	optimization run
EPI_ISL_18204844	B	optimization run
EPI_ISL_18204821	B	optimization run
EPI_ISL_18204852	B	optimization run
EPI_ISL_18204796	B	optimization run
EPI_ISL_18204760	A	optimization run
EPI_ISL_18204825	B	optimization run
EPI_ISL_18204873	B	optimization run
EPI_ISL_18204819	B	optimization run
EPI_ISL_18204878	B	optimization run
EPI_ISL_18277069	A	optimization run
